# Relationship of EMG/SMG features and muscle strength level: an exploratory study on tibialis anterior muscles during plantar-flexion among hemiplegia patients

**DOI:** 10.1186/1475-925X-13-5

**Published:** 2014-01-27

**Authors:** Huihui Li, Guoru Zhao, Yongjin Zhou, Xin Chen, Zhen Ji, Lei Wang

**Affiliations:** 1 Shenzhen Institutes of Advanced Technology, Chinese Academy of Sciences, Shenzhen, China; 2 Shenzhen Key Lab for Low-cost Healthcare, Shenzhen, China; 3 Shenzhen University, Shenzhen, China

**Keywords:** Sonomyography, Tibialis anterior muscle, Surface electromyography, Muscle strength level

## Abstract

**Background:**

Improvement in muscle strength is an important aim for the rehabilitation of hemiplegia patients. Presently, the rehabilitation prescription depends on the evaluation results of muscle strength, which are routinely estimated by experienced physicians and therefore not finely quantitative. Widely-used quantification methods for disability, such as Barthel Index (BI) and motor component of Functional Independent Measure (M-FIM), yet have limitations in their application, since both of them differentiated disability better in lower than higher disability, and they are subjective and recorded in wide scales. In this paper, to explore finely quantitative measures for evaluation of muscle strength level (MSL), we start with the study on quantified electromyography (EMG) and sonomyography (SMG) features of tibialis anterior (TA) muscles among hemiplegia patients.

**Methods:**

12 hemiplegia subjects volunteered to perform several sets of plantar-flexion movements in the study, and their EMG signals and SMG signals were recorded on TA independently to avoid interference. EMG data were filtered and then the root-mean-square (RMS) was computed. SMG signals, specifically speaking, the muscle thickness of TA, were manually measured by two experienced operators using ultrasonography. Reproducibility of the SMG assessment on TA between operators was evaluated by non-parametric test (independent sample T test). Possible relationship between muscle thickness changes (TC) of TA and muscle strength level of hemiplegia patients was estimated.

**Results:**

Mean of EMG RMS between subjects is found linearly correlated with MSL (R^2^ = 0.903). And mean of TA muscle TC amplitudes is also linearly correlated with MSL among dysfunctional legs (R^2^ = 0.949). Moreover, rectified TC amplitudes (dysfunctional leg/ healthy leg, DLHL) and rectified EMG signals (DLHL) are found in linear correlation with MSL, with R^2^ = 0.756 and R^2^ = 0.676 respectively. Meanwhile, the preliminary results demonstrate that patients’ peak values of TC are generally proportional to their personal EMG peak values in 12 dysfunctional legs and 12 healthy legs (R^2^ = 0.521).

**Conclusions:**

It’s concluded that SMG could be a promising option to quantitatively estimate MSL for hemiplegia patients during rehabilitation besides EMG. However, after this exploratory study, they should be further investigated on a larger number of subjects.

## Background

Hemiparesis, or muscular weakness of the upper and lower legs contralateral to the brain lesion, is the most frequent sign following a stroke or intracerebral haemorrhage (ICH) [1]. The severity of paresis post stroke is an important consideration since it is a common manifestation of stroke that relates to functional capabilities, discharge destination and mortality [2]. Therefore, improvement in muscle function is an important aim during the rehabilitation of hemiplegia patients. The rehabilitation prescription depends on the evaluation results, which are routinely estimated by experienced physicians.

At present, there are many clinical evaluation methods of disability measure for stroke patients with hemiplegia, such as Fugle-Meyer method [3], Bobath level method, National Institute of Health (NIH) stroke scale [4], Scandinavian Scale [5], Brunnstrom stage method and so on [6]. The measures of motor performance that they incorporate do not challenge muscles sufficiently to indicate their strength accurately [7]. Researchers or practitioners may have difficulties in interpreting the clinical meaning of the scores or changes in scores. Moreover, different physicians may make different evaluation decisions because the evaluation process is not finely quantitative. Additionally, the physicians can’t make individualized prescription due to the lack of detailed movement information [7].

To quantify disability in patients, activities of daily living (ADL) (Barthel Index, BI, and motor component of Functional Independent Measure, M-FIM) and categorical disability measures (Modified Rankin Scale, MRS) are also used [8]. Although used globally, the BI and M-FIM scales have limitations in their applications [8]. Yu et al. [7] presents Brunnstrom stage automatic evaluation for stroke patients using extreme learning machine. The real time movement data is collected by using 3-axis accelerometer sensors which are placed in the geometric center of arms.

Muscle strength testing has been used to monitor status and recovery of hemiplegia patients [9]. Considerable evidences suggest that the ability to perform a physical task is determined by a threshold level of muscular strength and endurance [10,11]. A decline in functional status is determined at least in part by muscle strength, flexibility, range of motion, physical fitness, and body composition [10,12]. Although older cohorts have the highest risk of developing disability, the association between muscular strength and endurance and the subsequent prevalence of functional limitations found in [13] indicates that this relationship persists even among middle-aged adults. The findings suggest that maintenance of strength throughout the lifespan may reduce the prevalence of functional limitations.

A widely-used tool to quantify disability in hemiplegia patients is EMG. Many studies have been previously reported on the relationship between the surface electromyogram (EMG) and muscle force [14-16], length [17], wrist joint torque [18], etc. Most studies relating the surface EMG to muscular force suggest that the amplitude of the EMG should increase proportionately with the square root of the tension, rather than linearly, if motor units fire independently of one another [19]. Some reports found that mean force-EMG relationships were highly non-linear but similar in shape for different cats and different speeds of locomotion [16]. But some authors have concluded that the magnitude of the EMG signal is directly proportional to muscle strength for isometric and/or isotonic contractions with constant speed for various muscles. Direct experiments have shown a linear relationship [20,21]. The force and integrated EMG relationship averaged across subjects showed almost the same linear correlation in spite of different electrode locations [22]. No linear correlation was observed between median power frequency of EMG and the knee extension force [22]. Although surface EMG is used to quantify muscle activity, the relationship between force and surface EMG during voluntary contractions is not fully understood [23]. EMG has been shown to be a useful method of evaluating patients with stroke and other neurological disorders [24,25], but factors that prevent the direct quantification of muscle force from EMG signal include cross-talk [26], variations in the location of the recording electrodes [27] and the involvement of synergistic muscles in force generation. And it’s difficult for surface EMG to detect the deep muscles non-invasively, due to the fact that the deep muscle EMG may be attenuated more and mixed by the superficial muscle EMG when reaching the skin surface [28].

Ultrasound is capable of visualizing normal and pathological muscle movements [29-42]. Ultrasound appeared to be even more sensitive in detecting fasciculations compared to EMG and clinical observations, because it can visualize a large muscle area and deeper located muscles [29,41]. Neuromuscular disorders give rise to structural muscle changes that can be visualized with ultrasound: atrophy can be objectified by measuring muscle thickness, while infiltration of fat and fibrous tissue increase muscle echo intensity [29]. Therefore, besides EMG, there is a newly prevailing means to quantify disability in patients, named as sonomyography (SMG). SMG [30], defined as the muscle thickness change detected from the real-time ultrasound images was related to the morphological change of the muscle during its contraction. SMG has been employed for the control of prosthesis [30], the assessment of muscle fatigue [31], isometric muscle contraction [32], dynamic muscle contraction [33], one dimensional SMG tracking the guided patterns of wrist extension [28], the relationship between torque and rectus femoris muscle morphological change in healthy adults [34], estimation and visualization of longitudinal muscle motion [35] and so on. However, there is a lack of research using SMG to investigate the relationship between thickness change (TC) of tibialis anterior (TA) muscles and muscle strength level (MSL) hemiplegia patients.

The purpose of this study was to investigate the relationship between TA muscle’s EMG/SMG features and MSL during plantar-flexion contraction. The relationship between EMG and SMG features was also studied.

## Methods

### Subjects

Some patients who had cerebral surgery and came to the physical therapy rehabilitation center within one month volunteered to participate in the experiment in Guangzhou Zhujiang Hospital, Guangdong, China. The study was approved by the institutional review board of Shenzhen Institutes of Advanced Technology, Chinese Academy of Sciences.

Enrolment was fulfilled the following inclusion criteria: (1) stable vital signs within 1 month; (2) cerebral infarction (CI) and intracerebral haemorrhage were verified by computed tomography angiography (CTA), magnetic resonance angiography (MRA) or magnetic resonance imaging (MRI); (3) patient was at the stage for post CI/ICH rehabilitation admission in the hospital. The exclusion criteria included: (1) cognitive disorder which made patient incapably follow the guidance of examiner; (2) severe motor dysfunction required contact guard; (3) paraplegia & quadriplegia; (4) course of CI/ICH over 3 weeks; (5) motor dysfunction diagnosis before the onset of CI/ICH; (6) patient required ankle-foot orthotic; (7) any physical complaints impeded the test, such as muscle pain.

Consent forms were obtained from the patients and his/her authorized legal representative. November-December 2012, a group of 27 patients were in the physical therapy rehabilitation center for post CI/ICH rehabilitation. 12 patients fulfilled conditions and participated in the study (9 male, 3 female, age 52.2 ± 11.1 years). The number of dysfunctional left legs of patients is 6, and the number of dysfunctional right leg is 6. The general information of patients in different muscle strength level (MSL) is listed in Table 1.

**Table 1:** Patients medical record in Guangzhou Zhujiang Hospital

**Muscle Strength Level (MSL)**	**Number of subjects**	**Number of dysfunctional legs (left/right)**
1	1	1/0
2	3	2/1
3	2	1/1
4	5	1/4
5	13	1/0

### Experimental procedure

Patient seated on an anchored wheel chair performed ankle joint dorsiflexion following the direction of examiner. The movement was completed under the command of examiner. Patient performed dorsiflexion with no force restriction. The excessive knee and hip joint movement were prohibited during plantar-flexion. Practice was carried out in ahead of test for patient knowing the start position, lift and lower action in dorsiflexion. The qualified movement was ensured in practice. Any undesired movements including overactivated/depressed activity and ankle eversion/inversion in dorsiflexion was corrected by examiner. If the undesired movement was caused by CI/ICH, it would be cancelled without any correction.

**Figure 1:**
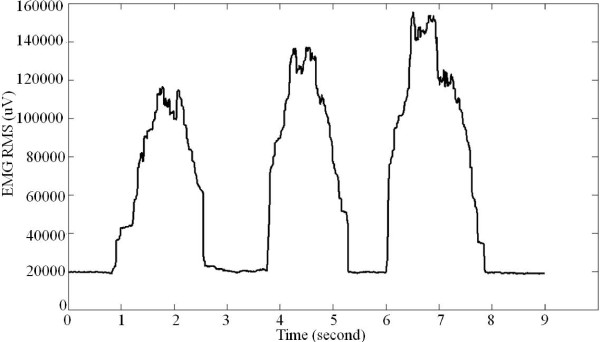
Patient’s EMG signal acquirement and ultrasound acquisition.

a) Start position: Patient kept the sole of foot on the floor and hold leg perpendicular with the floor (Figure 1).

b) Lift action: First activity (begin with the tone of ‘start’) to lift the sole of foot away from the ground while heel was inactivated on the floor. During this action, patients perform the maximal voluntary contraction (MVC) of the feet.

c) Lower action: Second activity (begin with the tone ‘stop’) of the foot back to the ground.

At least three consecutive trials should be completed. A two minutes rest was given after practice to avoid muscle fatigue in the test. Root mean square (RMS) of EMG was used to check the muscle activity. The qualified signal was recorded by the examiner.

### EMG assessment

Subject was seated in a chair with the knee flexed at 90 degrees and the ankle at neutral position. The surface EMG signals were recorded from the TA of right leg (channel 1), the TA of left leg (channel 2), the gastrocnemius of right leg (channel 3) and gastrocnemius of left leg (channel 4) by four pairs of surface electrodes (Figure 1). Each electrode (BioNomadix 2-Channel EMG system, BIOPAC Systems, Inc., Goleta, CA, USA) was separated from the other by 20 mm. Sampling rate was set at 1000 samples per second (1 KHz). The fixed gain of 2000 and 10 Hz high pass, 500 Hz low pass and notch filter off were preset for filter option. Noise voltage was 1.5 μV rms with bandwidth of 1.0 Hz to 500 Hz. The EMG data were exported as a MATLAB file and analyzed for peak value. EMG signal was quantified from RMS with interval of 0.5 s.

Maneuvers were carried out three times each, with the patient sat in a comfortable examination desk. Subjects were asked to initiate voluntary motor task movements under commands from the examiner for the duration of the flexion movement.

### SMG assessment

**Figure 2:**
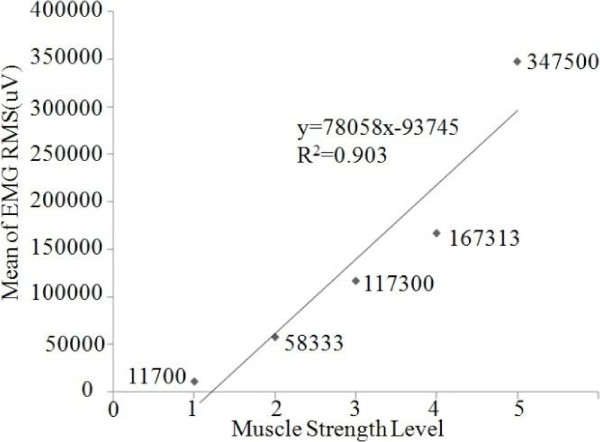
Profile of TA ACSA and TA thickness change (AB).

SMG assessment was not synchronized with EMG to avoid interference. Linear array ultrasound (US) transducer (Sonostar Technologies Co., Guangzhou, Guangdong, China) was placed perpendicular to the TA between the site of EMG ‘-’ and ‘+’ electrodes for transverse imaging measurement (Figure 1). The transducer was held properly by examiner to ensure consistent images in the test. Activity was following the command so that examiner could hold the position of probe on target. Besides, adequate coupling gel was used on transducer to guarantee sound wave well travel through the skin. Frequency of US transducer was at 7.5 MHz with 70 mm depth detection. Anatomical cross-sectional area (ACSA) was shown in Figure 2. Focus, contrast and brightness were adjusted for different imaging. In the trial, 128 frames within 10 seconds were collected for SMG analysis.

Images were manually measured in matlab (The MathWorks Inc., Natick, MA, USA). Image was cropped to a suitable size and analyzed in the sequence of frames. The TA ACSA was delineated by using solid white line and separated by muscle fascia boundary and the other tissues’ architecture in the images. Two examiners had been trained and experienced on muscle architecture finding in US image independently and were blinded from the profiles of patients. The difference between maximum thickness and minimum thickness of TA muscle thickness AB as showed in Figure 2 were measured twice. AB was recorded with pixel numbers of 6 per (mm). Thickness change (TC) between TA muscle in activity period (AP) and TA muscle in rest period (RB) was calculated by (1) of

(1) $$\mathrm{TC}=\left(AB_{\text{maximum}}‒AB_{\text{minimum}}\right)/6$$

AB ranging was admitted to visually differentiate the AP and RB by manual plot of continuous US images. The AP and RB periods were selected by examiner through observation that there were no paired points with the increasing and decreasing tendency indicating another AB ranging period in AP or RB.

### Statistical analysis

Statistical analysis was carried out in the SPSS version 19.0 software. Non-parametric test was applied to analyze SMG ACSA. Reproducibility of the SMG assessment on TC between two operators was evaluated by non-parametric test (independent sample T test). Two experienced examiners assessed each image two times in two different days.

## Results

### EMG analysis

**Figure 3:**
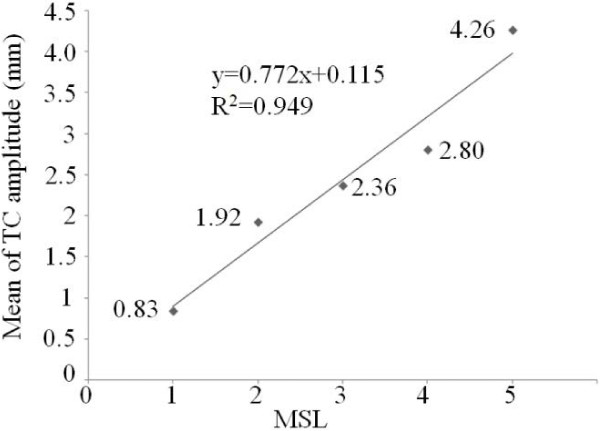
EMG RMS of patient during plantar-flexion movement (three times contractions).

**Figure 4:**
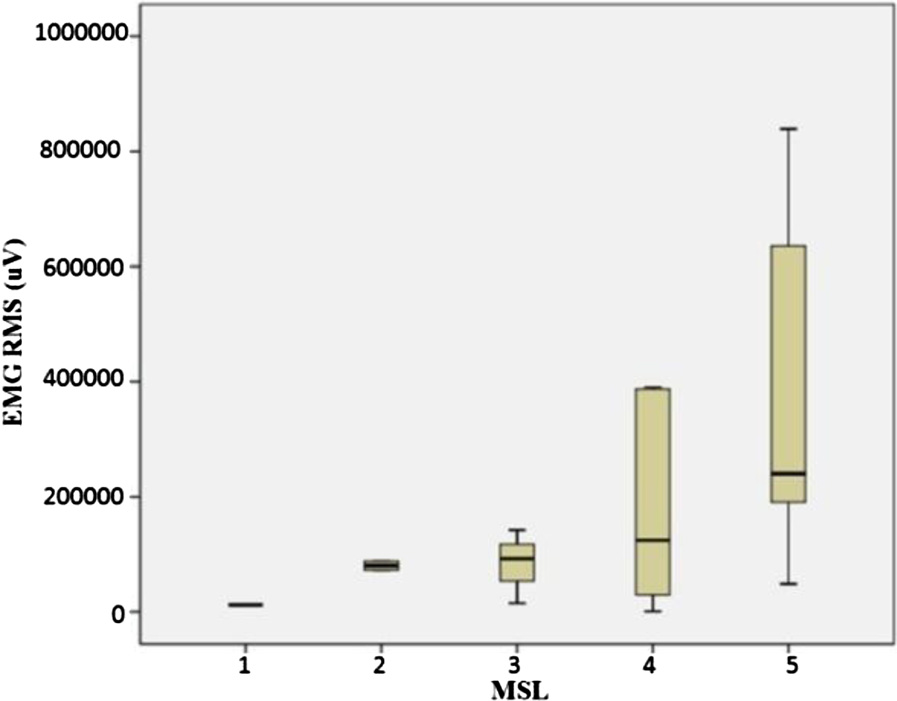
Mean of different patients’ EMG amplitudes versus muscle strength level.

Excel 2007 was used for the EMG analysis and SMG analysis. The EMG signal of patient was recorded during three-times plantar-flexion movements (Figure 3). It was found that mean of patients’ EMG amplitudes in each scale of MSL was proportional to MSL (Figure 4). The relationship for EMG RMS versus MSL was best-fitted with linear model (R^2^ = 0.903).

### SMG analysis

To investigate the significant difference between two examiners’ TC results, two group samples from different examiners were assessed by independent sample T test result. Statistical significance was accepted at the 5% level. There was no significant difference between two operators’ results (p > 0.1).

**Figure 5:**
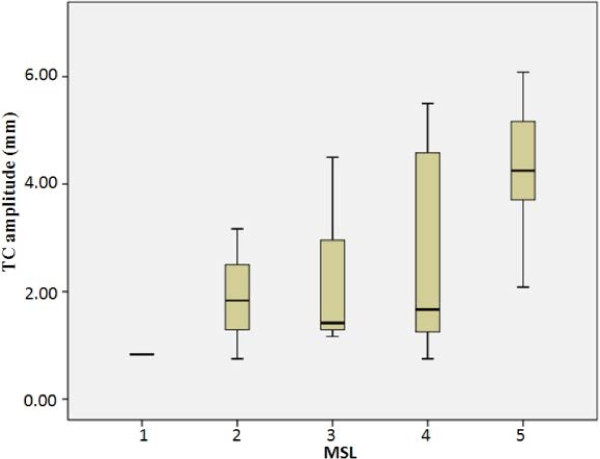
The relationship between the mean of TC amplitude and MSL.

To study the relationship between mean of TA TC in different hemiparetic legs and MSL, we computed the mean values of TA TC in each scale of MSL. It was observed in Figure 5 that mean of TC amplitude of patients was in good correlation with the MSL (R^2^ = 0.949). Mean of TC amplitude of hemiplegia legs increased almost linearly as the MSL increased.

**Figure 6:**
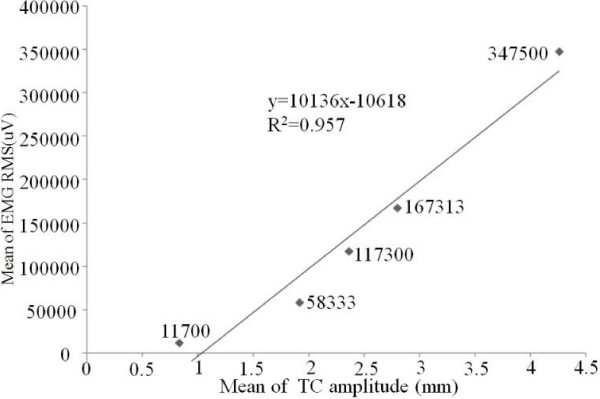
Boxplot of EMG RMS with peak value.

**Figure 7:**
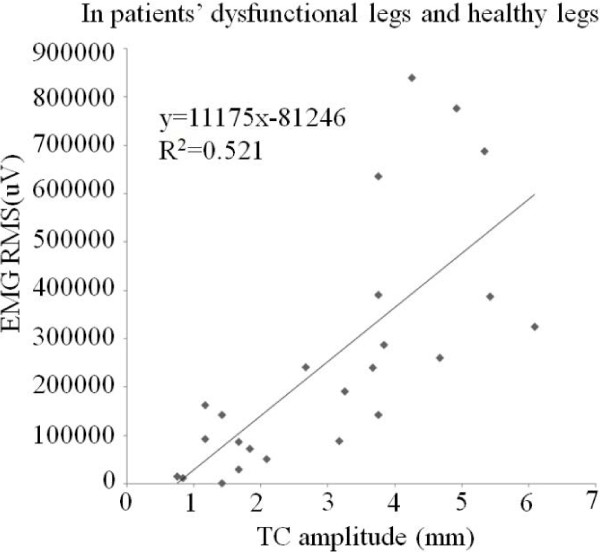
Boxplot of the mean of patients’ TC amplitudes and MSL.

At the same time, variances of EMG and SMG for all subjects in the same MSL were found large. Figure 6 and Figure 7 show boxplots of EMG RMS with peak value in the same MSL and the mean of TC amplitudes in the same MSL respectively.

**Figure 8:**
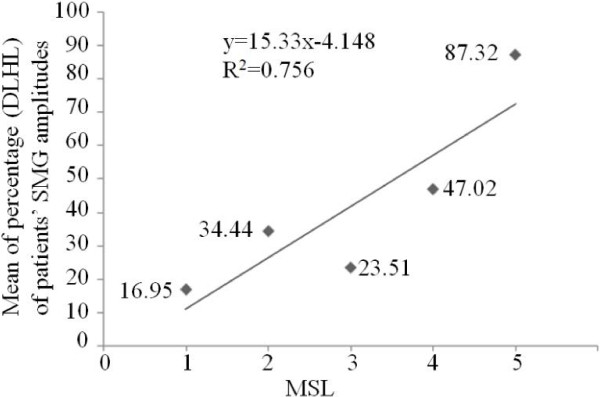
The mean values of EMG amplitudes and TC amplitudes.

**Figure 9:**
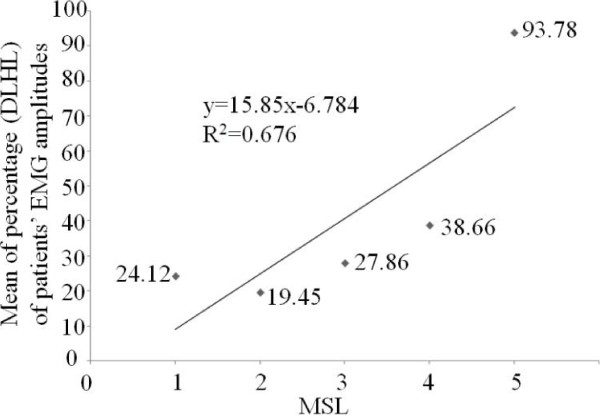
EMG amplitudes and corresponding TC amplitudes in 12 dysfunctional legs and 12 healthy legs.

In order to explore the relationship between mean of EMG RMS and mean of TC amplitude, a chart of the mean of EMG amplitudes and corresponding TC amplitudes is shown in Figure 8. A very high linearly correlation could be concluded between these two quantified parameters (R^2^ = 0.957). Furthermore, to study individual patient’s relationship between EMG signal and SMG signal, all EMG amplitudes and TC amplitudes (R^2^ = 0.521) in 12 dysfunctional legs and 12 healthy legs were shown in Figure 9. There is a nearly linear relationship between the EMG amplitude and TC amplitude for each patient. EMG amplitudes and TC amplitudes in 12 dysfunctional legs have a higher linear relationship (R^2^ = 0.686). Notice that it indicated that each patient’ inherent correlation between the EMG signal and TC signal in dysfunctional leg.

**Figure 10:**
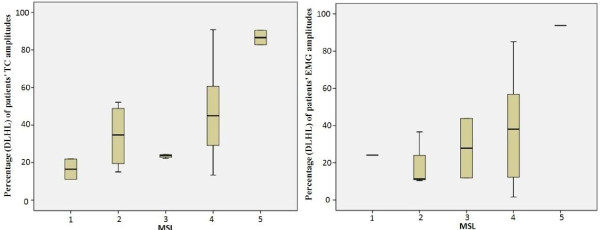
The relationship between the mean of rectified TC amplitudes (DLHL) and MSL.

**Figure 11:**
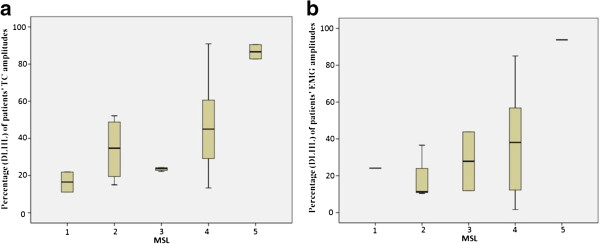
The relationship between the mean of rectified EMG amplitudes (DLHL) and MSL.

**Figure 12:**
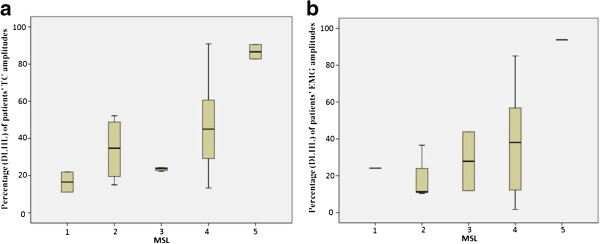
Boxplot of percentage (DLHL) (a) of patients’ TC amplitudes, (b) of patients’ EMG amplitudes.

Considering the individual difference among hemiplegia patients, a method was to be explored using the value of TC result in dysfunctional leg normalized by TC in healthy leg of same subject. The intercept of the linear regression of percentage (dysfunctional leg/healthy leg, DLHL) was used to calculate the representative value. Rectified TC amplitudes (DLHL) were proportional to MSL as shown in Figure 10. The relationship of rectified TC amplitudes (DLHL) and MSL was fitted with a linear model (R^2^ = 0.756). Moreover, mean of rectified EMG RMS amplitudes (DLHL) versus MSL was studied. There was a similar relationship between rectified EMG RMS amplitudes (DLHL) and MSL (R^2^ = 0.676) in Figure 11. Figure 12a and Figure 12b show boxplot of rectified TC amplitudes (DLHL) and rectified EMG amplitude (DLHL), respectively.

Based on the results from Figures 4, 5, 6, 7, 8 we presented reasonable hypothesis 1 that TC of patient in dysfunctional leg was smaller than that of patient in healthy leg. Given the hypothesis 1 exists, decisions are made to differentiate the dysfunctional leg and healthy leg with the TC value. Prediction results of the evaluation theme are shown in Table 2. The accuracy is 83.3% and 91.7% for operator 1 and operator 2’s result, respectively.

**Table 2:** Prediction results of the evaluation theme based on hypothesis 1

**Operator**	**The number of correct_predicted subjects**	**The number of wrong_predicted subjects**	**Accuracy (%)**
#1	10	2	83.3
#2	11	1	91.7

## Discussion

The report on TC during plantar-flexion among hemiplegia subjects is limited, therefore in this study, we engaged two experienced operators to process the US images manually. Though there were no statistical differences between their outputs, and we have taken the averaged values from both operators as TC, it should be noted the inter-operators difference is not totally trivial, 0.52 ± 1.39 mm.

Generally speaking, as shown in Figures 4 and 5, results of both EMG, as a popular means of muscle study, and SMG (TC of TA), are in strong correlation to the clinically prevailing MSL, which provided proofs to the assumption that the MSL can also be reflected muscle bio-electric activities and morphological changes.

However, it should be noted that in this experiments, large inter-subjects variances were observed for both EMG and SMG. For EMG, it has previously reported that the variance of EMG may provide additional information that is “lost” when using only an average EMG amplitude as EMG variance may indicate how the average muscle activity is occurring [43]. And fortunately, EMG and SMG themselves demonstrated good correlation, as shown in Figure 8, though they represented two distinct genres of signals generated during the muscle contractions. Furthermore, for individual patients, EMG amplitudes and corresponding TC amplitudes also had a linear relationship (as shown in Figure 9) in totally 12 dysfunctional and 12 healthy legs.

If we excluded the possible measurement errors, a major cause of the large inter-subjects variances therefore can be the variances of execution of the exercises. Specifically speaking, the differences of subjects in terms of both cognitive status and level of willing to wholly complete the exercises, as hardly controllable in an objective way, could lead to the large inter-subjects variances of EMG or SMG. In other words, if a subject does not try his/her best of strength, a variance will be contributed to the ‘true’ relationship between this scale of MSL and EMG or SMG, but less possible the relationship between EMG and SMG themselves.

Another concern is the ‘true’ inter-subjects variance for each MSL. Hence as stated in the method section, we also evaluated the DLHL. A highly significant relationship between rectified TC amplitude (DLHL) and MSL was observed (Figure 10). And a highly significant relationship between rectified EMG amplitude (DLHL) and MSL was observed (Figure 11).

The results of the experiments to differentiate dysfunctional leg and healthy leg using the TC showed a very good performance, as shown in Table 2, thus provided preliminary supports to hypothesis 1.

Previous research had shown that deficits in EMG area were not observed in all paretic muscles. The type of contraction influenced the degree of deficits demonstrated in the paretic muscles, with greater preservation of force during eccentric contractions [44]. The EMG of the paretic TA could be used as a control signal for the beginning of electrical stimulation, through the detection of changes in their RMS values during the hemiparetic gait [45] and TA EMG data could be used in sleep/wake estimation [46]. The reason we choose TA as the object muscle in this study is, TA is independent relatively and less possibly affected by other muscles during the plantar-flexion movement. Yet large variance existed in this study for the TA EMG signals as shown before, and the reasons remain unclear and require further investigations.

## Conclusions

In an exploratory study among the hemiplegia patients, both mean of EMG RMS in each MSL scale for TA and mean of TA TC in each MSL scale during plantar-flexion were strongly correlated with MSL. There was a good correlation between mean of EMG amplitudes and TC amplitudes as well. There was a nearly linear relationship between the EMG amplitude and TC amplitude for each patient among dysfunctional legs. Results from the current study provided preliminary insights to the quantitative relationship between EMG/SMG and MSL as well as EMG and SMG themselves, in an example muscle of TA producing the plantar-flexion exercises among hemiplegia patients. Thus it suggests that SMG could provide an alternative perspective to understand the muscle contraction activity morphologically among hemiplegia patients, along with the more well-reported EMG. Further investigations on how such morphological information could be applied to evaluation of muscle function, yet require experiments on a larger number of subjects with further clustering according to factors such as age, gender or pathological origins.

## Abbreviations

BI: Barthel index; M-FIM: Motor component of functional independent measure; EMG: Electromyography; SMG: Sonomyography; TA: Tibialis anterior; RMS: Root mean square; MSL: Muscle strength level; TC: Thickness change; ICH: Intracerebral haemorrhage; NIH: National Institute of Health; ADL: Activities of daily living; MRS: Modified rankin scale; CTA: Computed tomography angiography; MRA: Magnetic resonance angiography; MRI: Magnetic resonance imaging; MVC: Maximal voluntary contraction; MAV: Mean absolute value; SD: Standard deviation; US: Ultrasound; ACSA: Anatomical cross-sectional area; AP: Activity period; RB: Rest period; DLHL: Dysfunctional leg/healthy leg.

## Competing interests

The authors declare that they have no competing interests.

## Authors’ contributions

HHL: analyzed the data with XC, ZJ and LW, and composed the manuscript together with GRZ and YJZ, YJZ: initiated the idea and deployed the experiments with GRZ. All authors read and approved the final manuscript.

## References

[B1] Chan CWY Motor and sensory deficits following a stroke: relevance to a comprehensive evaluation Physiother Can 1986 38 29 34

[B2] Andrews AW Bohannon RW Distribution of muscle strength impairments following stroke Clin Rehabil 2000 14 79 87 10.1191/026921500673950113 10688348

[B3] Fugl-Meyer AR Jaasko L Leyman I Olsson S Steglind S The post-stroke hemiplegic patient: a method for evaluation of physical performance Scand J Rehabil Med 1975 7 13 31 1135616

[B4] Goldstein LB Bertels C Davis JN Interrater reliability of the Nih stroke scale Arch Neurol-Chicago 1989 46 660 662 10.1001/archneur.1989.00520420080026 2730378

[B5] Group SSS Multicenter trial of hemo dilution in ischemic stroke-background and study protocol Stroke 1985 16 885 890 3901425 10.1161/01.str.16.5.885

[B6] Guo YB Wang EY Cai LH Application of quality of life scale in stroke Chin J Rehabil Theory Pract 2009 15 632 634

[B7] Yu L Wang JP Fang Q Wang Y Brunnstrom stage automatic evaluation for stroke patients using extreme learning machine IEEE BioCAS Conf 2012 Hsinchu, Taiwan: IEEE 380 383

[B8] Kwon S Hartzema AG Duncan PW Lai SM Disability measures in stroke - relationship among the barthel index, the functional independence measure, and the modified rankin scale Stroke 2004 35 918 923 10.1161/01.STR.0000119385.56094.32 14976324

[B9] Richard WB Melissa BS Assessment of strength deficits in eight paretic upper extremity muscle groups of stroke patients with hemiplegia Phys Ther 1987 67 522 525 3562543 10.1093/ptj/67.4.522

[B10] Brown M Sinacore DR Host HH The relationship of strength to function in the older adult J Gerontol a-Biol 1995 50 55 59 10.1093/gerona/50a.special_issue.55 7493219

[B11] Buchner DM Preserving mobility in older adults Western J Med 1997 167 258 264 PMC1304541 9348757

[B12] Hyatt RH Whitelaw MN Bhat A Scott S Maxwell JD Association of muscle strength with functional status of elderly people Age Ageing 1990 19 330 336 10.1093/ageing/19.5.330 2251967

[B13] Brill PA Macera CA Davis DR Blair SN Gordon N Muscular strength and physical function Med Sci Sport Exer 2000 32 412 416 10.1097/00005768-200002000-00023 10694125

[B14] Hof AL EMG and muscle force: an introduction Human Movement Sci 1984 3 119 153 10.1016/0167-9457(84)90008-3

[B15] Thongpanja AP S Phukpattaranout P Limsakul C Mean and median frequency of EMG signal determine muscle force based on time-dependent power spectrum Electronics and Electrical Engineering 2013 19 51 56

[B16] Guimaraes AC Herzog W Allinger TL Zhang YT The Emg-force relationship of the Cat soleus muscle and its association with contractile conditions during locomotion J Exp Biol 1995 198 975 987 7730759 10.1242/jeb.198.4.975

[B17] Mannion AF Dolan P The effects of muscle length and force output on the EMG power spectrum of the erector spinae Human J Electromyogr Kinesiol 1996 6 159 168 10.1016/1050-6411(95)00028-3 20719673

[B18] Lee J Kagamihara Y Kakei S Quantitative evaluation of movement disorders in neurological diseases based on EMG signals30th Annual International IEEE EMBS Conference; August 20-24 2008 Vancouver, British Columbia, Canada: IEEE 10.1109/IEMBS.2008.4649120 19162623

[B19] Shwedyk E Balasubramanian R Scott RN A nonstationary model for the Electromyogram IEEE Trans Biomed Eng 1977 24 417 424 892834 10.1109/TBME.1977.326175

[B20] Bohannon RW Larkin PA Smith MB Horton MG Relationship between static muscle strength deficits and spasticity in stroke patients with hemiparesis Phys Ther 1987 67 1068 1071 3602099 10.1093/ptj/67.7.1068

[B21] Perry J Bekey GA Emg-force relationships in skeletal-muscle Crit Rev Biomed Eng 1981 7 1 22 7042199

[B22] Onishi H Yagi R Akasaka K Momose K Ihashi K Handa Y Relationship between EMG signals and force in human vastus lateralis muscle using multiple bipolar wire electrodes J Electromyogr Kines 2000 10 59 67 10.1016/S1050-6411(99)00020-6 10659450

[B23] Kuriki HU Takahashi LSO Schwartz M Relationship between electromyography and muscle force EMG Methods for evaluating muscle and nerve function 2012 http://www.intechopen.com/download/get/type/pdfs/id/25852

[B24] Sabut SK Lenka PK Kumar R Mahadevappa M Effect of functional electrical stimulation on the effort and walking speed, surface electromyography activity, and metabolic responses in stroke subjects J Electromyogr Kines 2010 20 1170 1177 10.1016/j.jelekin.2010.07.003 20692180

[B25] Arya BK Mohapatra J Subramanya K Prasad H Kumar R Mahadevappa M Surface EMG analysis and changes in gait following electrical stimulation of quadriceps femoris and tibialis anterior in children with spastic cerebral palsy IEEE EMBS Conf 2012 San Diego, CA, United states: IEEE 5726 5729 10.1109/EMBC.2012.6347295 23367230

[B26] DeLuca CJ Surface Electromyography: Detection and Recording Surface Electromyography: Detection and Recording 2002 Boston: DelSys Incorporated 1 10

[B27] Alemu M Kumar DK Bradley A Time-frequency analysis of SEMG - With special consideration to the interelectrode spacing Ieee T Neur Sys Reh 2003 11 341 345 10.1109/TNSRE.2003.819903 14960108

[B28] Guo JY Zheng YP Huang QH Chen X He JF Comparison of sonomyography and electromyography of forearm muscles in the guided wrist extension Proc 5th International workshop on Wearable and Implantable body sensor Networks 2008 HongKong, China: IEEE 235 238

[B29] Pillen S Skeletal muscle ultrasound Eur J Transl Myol 2010 1 145 155

[B30] Zheng YP Chan MMF Shi J Chen X Huang QH Sonomyography: monitoring morphological changes of forearm muscles in actions with the feasibility for the control of powered prosthesis Med Eng Phys 2005 28 405 415 16115790 10.1016/j.medengphy.2005.07.012

[B31] Shi J Zheng YP Chen X Huang QH Assessment of muscle fatigue using sonomyography: muscle thickness change detected from ultrasound images Med Eng Phys 2007 29 472 479 10.1016/j.medengphy.2006.07.004 16908212

[B32] Shi J Zheng YP Huang QH Chen X Continuous monitoring of sonomyography, electromyography and torque generated by normal upper arm muscles during isometric contraction: sonomyography assessment for arm muscles IEEE Trans Biomed Eng 2007 55 1191 1198 18334413 10.1109/TBME.2007.909538

[B33] Guo JY Zheng YP Huang QH Chen X Dynamic monitoring of forearm muscles using 1D sonomyography (SMG) system J Rehabil Res 2008 45 187 196 10.1682/JRRD.2007.02.0026 18566937

[B34] Chen X Zheng YP Guo JY Zhu ZY Chan SC Zhang ZG Sonomyographic responses during voluntary isometric ramp contraction of the human rectus femoris muscle Eur J Appl Physiol 2012 112 2603 2614 10.1007/s00421-011-2227-2 22081124 PMC3371332

[B35] Li JZ Zhou YJ Ivanov K Zheng YP Estimation and visualization of longitudinal muscle motion using ultrasonography: a feasibility study Ultrasonics 2014 54 779 788 10.1016/j.ultras.2013.09.024 24206676

[B36] Han P Chen Y Ao L Xie G Li H Wang L Zhou Y Automatic thickness estimation for skeletal muscle in ultrasonography: evaluation of two enhancement methods Biomed Eng Online 2013 12 doi:10.1186/1475-925X-12-6 10.1186/1475-925X-12-6 PMC3626569 23339544

[B37] Ling S Zhou Y Chen Y Zhao Y Wang L Zheng Y Automatic tracking of aponeuroses and estimation of muscle thickness in ultrasonography: a feasibility study IEEE J Biomed Health Inform 2013 11 6 1031 1038 24240721 10.1109/JBHI.2013.2253787

[B38] Ling S Chen B Zhou Y Yang W Zhao Y Wang L Zheng Y An efficient framework for estimation of muscle fiber orientation using ultrasonography Biomed Eng Online 2013 12 98 doi:10.1186/1475-925X-12-98 10.1186/1475-925X-12-98 24079340 PMC3851156

[B39] Li J Zhou Y Lu Y Zhou G Wang L Zheng Y Sensitive and efficient detection of quadriceps muscle thickness changes in cross-sectional plane using ultrasonography: a feasibility investigation IEEE Trans Inf Technol Biomed 2013 doi:10.1109/JBHI.2013.2275002 10.1109/JBHI.2013.2275002 24608062

[B40] Zhou Y Li J Zhou G Zheng Y Dynamic measurement of pennation angle of gastrocnemius muscles during contractions based on ultrasound imaging Biomed Eng Online 2012 11 63 doi:10.1186/1475-925X-11-63 10.1186/1475-925X-11-63 PMC3776435 22943184

[B41] Zhou Y Zheng Y Longitudinal enhancement of the hyperechoic regions in ultrasonography of muscles using a Gabor filter bank approach: a preparation for semi-automatic muscle fiber orientation estimation Ultrasound Med Biol 2011 37 4 665 673 10.1016/j.ultrasmedbio.2010.12.011 21371811

[B42] Zhou Y Zheng Y Estimation of muscle fiber orientation in ultrasound images using revoting hough transform (RVHT) Ultrasound Med Biol 2008 34 9 1474 1481 10.1016/j.ultrasmedbio.2008.02.009 18420336

[B43] Rokicki LA Houle TT Dhingra LK Weinland SR Urban AM Bhalla RK A preliminary analysis of EMG variance as an index of change in EMG biofeedback treatment of tension-type headache Appl Psychophysiol Biofeedback 2003 28 205 215 10.1023/A:1024633230584 12964452

[B44] Gray VL Ivanova TD Garland SJ Effects of fast functional exercise on muscle activity after stroke Neurorehab Neural Re 2012 26 968 975 10.1177/1545968312437944 22412173

[B45] Bonell CE Cherniz AS Tabernig CB Analysis of EMG temporal parameters from the tibialis anterior during hemiparetic gait16th Argentine Bioengineering Congress and the 5th Conference of Clinical Engineering 2007 San Juan, Argentina: Journal of Physics:Conference Series 1 6 doi:10.1088/1742-6596/90/1/012087

[B46] Hwang S Chung G Lee J Shin J Lee SJ Jeong DU Park K Sleep/wake estimation using only anterior tibialis electromyography data Biomed Eng Online 2012 11 1 15 10.1186/1475-925X-11-1 22624953 PMC3476968

